# Immediate effects of the Fukushima nuclear power plant disaster on depressive symptoms among mothers with infants: a prefectural-wide cross-sectional study from the Fukushima Health Management Survey

**DOI:** 10.1186/s12888-015-0443-8

**Published:** 2015-03-26

**Authors:** Aya Goto, Evelyn J Bromet, Kenya Fujimori, for the Pregnancy and Birth Survey Group of the Fukushima Health Management Survey

**Affiliations:** Department of Public Health, Fukushima Medical University School of Medicine, Hikarigaoka 1, Fukushima City, Fukushima 960-1295 Japan; Department of Psychiatry, State University of New York at Stony Brook, Putnam Hall - South Campus, Stony Brook, 11794-8790 NY USA; Department of Obstetrics and Gynecology, Fukushima Medical University School of Medicine, Hikarigaoka 1, Fukushima City, Fukushima 960-1295 Japan

**Keywords:** Mothers, Depression, Maternal health services, Radiation, Fukushima nuclear accident, Japan

## Abstract

**Background:**

Mothers of young children are at high-risk for developing adverse mental health effects following a nuclear accident. Using the Japanese pregnancy registration system, the prefecture of Fukushima launched a population-based survey of women who were pregnant at the time of the Fukushima nuclear accident in order to assess their and their newborns’ health. In this paper, we focus on the results of a screen for depressive symptoms among new mothers and its association with geographical region and interruption of obstetrical care after the Fukushima nuclear accident, which occurred after the Great East Japan Earthquake on March 11, 2011.

**Methods:**

The survey targeted women who lived in Fukushima prefecture and who had registered their pregnancies between August 1, 2010 and July 31, 2011. Among the 16,001 women targeted, 9,321 returned the questionnaires (response proportion = 58.3%) and data from 8,196 women with singleton live births were analyzed. The main outcome measure was a standard two-item depression screen. Regional radiation levels were determined from the prefecture’s periodical reports, and interruption in obstetrical care after the Fukushima nuclear accident was determined from mothers’ individual responses to the questionnaire.

**Results:**

Among the 8,196 women, 2,262 (28%) screened positive for depressive symptoms. After adjusting for maternal and infant characteristics, both mothers in Soso, the region in which the nuclear power plant is located, and mothers that had changed obstetrical care facilities were significantly more likely to screen positive for depression. In contrast, mothers in Iwaki and Aizu, regions with relatively low radiation levels, were significantly less likely to screen positive for depression.

**Conclusions:**

Our findings suggest that improving mental health support for mothers with infants should be a high priority in the acute phase of nuclear disaster response. We further recommend that in the strategic provisioning of parental support, close attention should be paid to regional variations in negative mental health consequences, particularly to those who experienced an interruption in their obstetrical care.

## Background

The mental health consequences of human-made toxicological disasters, including nuclear accidents, appear to be more enduring than those that occur following natural disasters [1]. Mothers of young children are one of the groups at greatest risk for negative emotional responses and poorer mental health following a nuclear accident [2]. The Great East Japan Earthquake, which occurred on March 11, 2011, was a multiple disaster that included an earthquake, tsunami, and a massive nuclear power plant accident that caused immediate disruption to medical care [3] and persistent radiation contamination across a broad geographic area [4].

According to a report presented at the annual meeting of the Japan Association of Obstetricians and Gynecologists [5], four childbirth facilities (out of 50 facilities in the prefecture) were shut down during the first month after the disaster. These four facilities were located in the coastal area that was most affected by the triple disasters, and the shutdown resulted in six women having to be referred to the only university hospital in the prefecture. This hospital is located in the capital, Fukushima City, approximately 70 km inland. While the university hospital was initially without water for one week, it was able to maintain its basic functions and the Department of Obstetrics and Gynecology received nine referrals for cesarean sections in the first ten days following the disaster: six from the affected coastal area and three from within the city [6].

**Figure 1:**
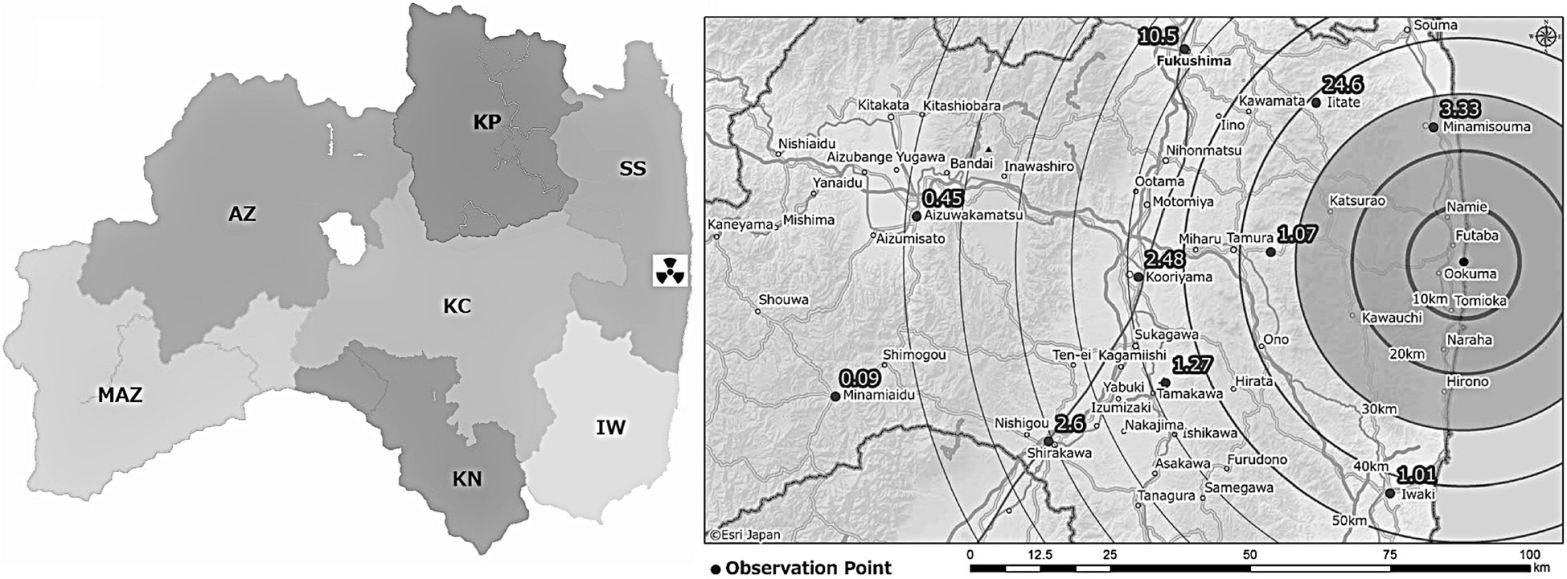
Seven regions in Fukushima prefecture and radioactivity measurements taken on March 18, 2011*. From the right side on the map: SS: Soso, IW: Iwaki, KP: Kenpoku, KC: Kenchu, KN: Kennan, AZ: Aizu, MAZ: Minamiaizu. * Radioactivity measurements were originally published in Nagata et al. [7], reproduced with publisher permission (License Number 3365080379769).

A large area in Soso, the region in which the damaged nuclear power plant is located, was designated as an evacuation zone due to environmental radiation contamination. The radioactive plume from the nuclear power plant was carried north by the wind toward the prefecture’s most populated region, Kenpoku [7]. As a result, the capital city in Kenpoku had the second-highest level of radiation of all the areas assessed by the Geographic Information System, as reported by Nagata and colleagues and presented here in Figure 1. In contrast, the mountainous regions of Aizu and Minamiaizu recorded the lowest levels of radiation among these regions.

Our previous study on parent counseling records among mothers with young children in Fukushima City, an area with relatively high radiation contamination, found that changes in daily routine and concerns about radiation had a negative psychological impact [8]. We further found that risk for depressive symptoms was associated with interpersonal problems at home, as well as living in areas with higher radiation levels within the city [8,9]. The current analysis aims to extend these findings from the Fukushima City study by focusing on mothers across the prefecture.

Specifically, Fukushima Medical University launched a prefecture-wide cohort survey (Fukushima Health Management Survey; FHMS) within one year of the disaster to investigate the mental and physical health of the population, and to lay the foundation for future research on the potential effects of low-dose radiation exposure on health [10]. An initial basic survey was conducted to estimate levels of external radiation exposure. Four focused surveys were then administered, including the Pregnancy and Birth Survey that targeted women who were pregnant at the time of the nuclear accident. That survey built on the Japanese pregnancy registration system, the aim of which is to provide free access to antenatal care and well-child visits, and assessed the health of both mothers and their newborns. The current study examines regional variations in depressive symptoms among these women and associations with interruption in obstetrical care services after the Fukushima nuclear accident.

## Method

### Study design and subjects

The Pregnancy and Birth Survey of the FHMS has been conducted every year since the disaster. In the current study, we analyzed data derived from the very first survey in 2011, targeting women who lived in the Fukushima Prefecture, and who had registered their pregnancies from August 1, 2010 to July 31, 2011. Given that the earliest a woman can register her pregnancy in Japan is in the fourth gestational week, and the latest is at the time of delivery, this target period was expected to cover most women who were pregnant at the time of the Great East Japan Earthquake on March 11, 2011. Lists of women who had registered their pregnancies within the designated period were obtained from all municipalities in the prefecture. Among the 16,001 identified women, 9,321 returned the FHMS questionnaires (response proportion = 58.3%), although 59 were later deemed invalid and eliminated from the database (5 blank questionnaires, 1 deceased, 21 duplicated mails, and 32 non-eligible cases). In the present study, we further excluded another 1,066 women: 459 whose pregnancy ended before the disaster, 145 with an unknown date of delivery, nine that experienced more than one pregnancy during the target period, nine who had a delayed survey response of over ten months, 21 who were living outside Fukushima Prefecture, 191 whose pregnancy outcome was one other than a live birth, 76 who gave birth to twins, 54 whose questionnaires were completed by someone else, and 102 women with missing data that related to depression. Thus, the current sample included 8,196 women with singleton live births. The demographic characteristics of all respondents (including those eliminated from the present analysis) have been previously reported [11].

### Survey items

The survey included a two-item screening measure of depression [12], which inquired about feelings of depressed mood and anhedonia over the past month. The two items were “During the past month, have you often felt down, depressed, or hopeless?” and “During the past month, have you often found little interest or pleasure in doing things?” Mothers who answered yes to at least one of these questions were classified as displaying depressive symptoms. In a previous study of 103 Japanese mothers using the Edinburgh Postnatal Depression Scale (EPDS) as a gold standard, the sensitivity and specificity of the current screening measure was 88% and 76%, respectively [12].

Associations between depressive symptoms and the following variables were analyzed: maternal factors (age at pregnancy, gestational age at the time of disaster, number of postpartum days at the time of survey response, and psychiatric history); obstetrical factors (complications during pregnancy, mode of pregnancy and delivery, and gestational week at birth); and infant characteristics (sex, birth weight, asphyxia at birth, and other anomalies). Residential region and obstetrical care after the disaster were also included as independent factors of interest. Psychiatric history was self-reported in the questionnaire and included pre-pregnancy, during pregnancy, and postnatal occurrences. Likewise, obstetrical complications were self-reported using a list of 15 complications. Modes of pregnancy included natural pregnancy, ovulation induction, artificial insemination, and in vitro fertilization.

As explained above, residential region and obstetrical care after the disaster were a focus of the current study due to previous investigations reporting a higher frequency of depressive symptoms in areas that experienced higher radiation levels within one city [9], and more severe disruptions of obstetrical care immediately following the disaster [6]. Radiation levels were based on the hourly average, region-specific measurements taken in August 2011, as reported by the prefectural office [13]. Readings from August 2011 were used, as this was around the latest month in which our target population registered their pregnancies. With regard to obstetrical care, we asked women whether they changed the medical facility in which they were receiving antenatal and obstetrical care following the disaster. It is worthy of note that the Japanese health care and insurance systems do not limit patients’ choice of medical facility.

### Statistical analysis

In order to conduct a sensitivity analysis to assess the potential effect of response bias on the proportion of mothers that screened positive for depressive symptoms, we assumed that non-respondents would demonstrate the same prevalence of depressive symptoms as delayed responders. For the purpose of this study, delayed respondents were defined as those that responded to the survey only after a reminder had been sent out. This calculation was based on the approach of Meiklejohn and colleagues [14].

During the analyses of factors associated with depressive symptoms, we first used the Wilcoxon–Mann–Whitney rank sum test for continuous variables and chi-squared tests for categorical variables. These factors and subsequent associations are shown in Table 1.

**Table 1:** **Sample characteristics and differences by depressive symptoms**

	**N (%) or** ***Median (Min, Max)***			
**Characteristics**	**Total** ^**a**^ **(N = 8196)**	**Depressive symptoms**		**P value** ^**b**^
		**Positive (N = 2262)**	**Negative (N = 5934)**	
**Maternal characteristics**				
Mother’s age at the time of pregnancy (years)	***30 (15, 51)***	*30 (16, 45)*	*30 (15, 51)*	<0.01
Postpartum days at the time of survey (days)	***175 (0, 647)***	*179 (0, 599)*	*174 (0, 647)*	0.65
Gestational week at the time of disaster (weeks)	***19 (0, 41)***	*20 (0, 41)*	*19 (0, 41)*	0.13
Pregnancy history				
Birth				
0 (First-time mother)	**2248 (28.8)**	669 (29.8)	1579 (70.2)	
≥1	**5562 (71.2)**	1463 (26.3)	4099 (73.7)	<0.01
Miscarriage				
0	**2303 (61.0)**	619 (26.9)	1684 (73.1)	
≥1	**1475 (39.0)**	416 (28.2)	1059 (71.8)	0.37
Still birth				
0	**2808 (97.0)**	754 (26.9)	2054 (73.2)	
≥1	**86 (3.0)**	31 (36.1)	55 (64.0)	0.06
Abortion				
0	**2462 (70.5)**	642 (26.1)	1820 (73.9)	
≥1	**1032 (29.5)**	365 (35.4)	667 (64.6)	<0.01
Psychiatric history				
Before pregnancy				
No	**8071 (98.5)**	2187 (27.1)	5884 (72.9)	
Yes	**125 (1.5)**	75 (60.0)	50 (40.0)	<0.01
During pregnancy before the disaster				
No	**8111 (99.0)**	2209 (27.2)	5902 (72.8)	
Yes	**85 (1.0)**	53 (62.4)	32 (37.7)	<0.01
During pregnancy after the disaster				
No	**7877 (96.1)**	2075 (26.3)	5802 (73.7)	
Yes	**319 (3.9)**	187 (58.6)	132 (41.4)	<0.01
**Obstetrical characteristics**				
Mode of pregnancy				
Natural	**7841 (95.9)**	2178 (27.8)	5663 (72.2)	
Induced ovulation, or artificial insemination, in vitro fertilization	**333 (4.1)**	80 (24.0)	253 (76.0)	0.13
Mode of delivery				
Vaginal birth	**5570 (69.1)**	1507 (27.1)	4063 (72.9)	
Cesarean section, vacuum, or forceps	**2486 (30.9)**	714 (28.2)	1772 (72.3)	0.12
Gestational week at birth				
≥37	**7859 (96.1)**	2166 (27.6)	5693 (72.4)	
<37 (Preterm)	**317 (3.9)**	90 (28.4)	227 (71.6)	0.75
Obstetrical complications^c^ before the disaster				
No	**6920 (85.8)**	1809 (26.1)	5111 (73.9)	
Yes	**1154 (14.2)**	390 (34.1)	755 (65.9)	<0.01
Obstetrical complications^c^ after the disaster				
No	**6673 (85.7)**	1692 (25.4)	4981 (74.6)	
Yes	**1116 (14.3)**	360 (32.3)	756 (67.7)	<0.01
**Infant characteristics**				
Sex				
Girl	**3937 (48.4)**	1100 (27.9)	2837 (72.1)	
Boy	**4194 (51.6)**	1143 (27.3)	3051 (72.8)	0.49
Birth weight				
2500 g or higher	**7536 (92.6)**	2080 (27.6)	5456 (72.4)	
1500–2499 g	**564 (6.9)**	148 (26.2)	416 (73.8)	0.49
Less than 1500 g	**36 (0.4)**	15 (41.7)	21 (58.3)	0.06
Asphyxia				
No	**7812 (98.9)**	2152 (27.6)	5660 (72.5)	
Yes	**90 (1.1)**	35 (38.9)	55 (61.1)	0.02
Congenital anomaly				
No	**7693 (97.4)**	2098 (27.3)	5595 (72.7)	
Yes	**205 (2.6)**	86 (42.0)	119 (58.1)	<0.01

^a^Column proportions are shown for the total distribution.

^b^Wilcoxon–Mann–Whitney rank sum test was used for continuous variables and chi-squared tests were used for categorical variables.

^c^Excluding mental health disorders that are listed above in maternal psychiatric history.

The association between residential region and depressive symptoms was examined using unadjusted and adjusted logistic regression analyses. Four variables were significantly associated with both the outcome (depressive symptoms) and the pertinent independent variable (residential region): maternal age, birth history, psychiatric history during pregnancy after the disaster, and obstetrical complications after the disaster. Among them, psychiatric history during pregnancy and obstetrical complications after the disaster were considered to be potential mediators, and both maternal age and birth history were entered into the multivariate analysis. The Sobel-Goodman test was used to confirm the presence of a mediation effect of these two variables, and utilization of the Akaike information criterion confirmed that the two-factor model best fit the data.

Finally, the association between a change in medical facility and depressive symptoms was examined using the same procedure that was applied to identify confounders. Six variables were associated with both depression and change in facility: maternal age, psychiatric history during pregnancy after the disaster, obstetrical complications both before and after the disaster, infant’s asphyxia, and congenital anomaly. After excluding two potential mediators (psychiatric history during pregnancy and obstetrical complications after the disaster), the remaining four were included in the adjusted model along with residential region. In order to examine whether the relationship between any change in medical facilities and depression differed by the degree to which the local region was affected by the disaster, we repeated the same analysis and compared the severely affected coastal regions (Soso and Iwaki) with regions not as strongly exposed (Kenchu, Kenpoku, Kennan, and Aizu and Minamiaizu).

A p-value of less than 0.05 was considered as statistically significant. All statistical analyses were conducted via STATA, version 10.

### Ethical considerations

The ethics committee of Fukushima Medical University approved this study (No. 13048). The survey aims were explained to all respondents in a cover letter that was sent out with the questionnaire. By responding to the survey participants were considered to have consented to participation.

## Results

The median maternal age was 30 years and the median number of postpartum days at the time of the survey was 175. The proportion of infants with a low birth weight was 7.3%, while 2.6% were born with a congenital anomaly.

Among the 8,196 women surveyed, 27.6% screened positive for depressive symptoms. A sensitivity analysis based on the assumption that non-respondents would have similar prevalence to delayed responders estimated that the proportion would be 26.5%. Mothers with depressive symptoms were more likely to be young, first-time mothers, and with histories of abortion, psychiatric diseases, and complications during pregnancy, as well as infants with asphyxia or other anomalies (Table 1). The median time from delivery to returning the questionnaire was 175 days for all mothers.

Table 2 shows a wide regional variation in the proportion of mothers with depressive symptoms, with the highest rate in Soso (coastal region) and lowest in Aizu (mountainous region). After controlling for maternal age and birth history, it was found that Soso, the region in which the nuclear power plant is located, had a significantly higher proportion of mothers with depressive symptoms (aOR = 1.36, 95% CI = 1.15–1.62, p < 0.01). Conversely, Iwaki and Aizu, which had relatively lower radiation levels, had a significantly lower proportion of mothers with depressive symptoms (Iwaki: aOR = 0.84, 95% CI = 0.71–0.98, p = 0.03; Aizu and Minamiaizu: aOR = 0.72, 95% CI = 0.60–0.87, p < 0.01).

**Table 2:** **Regional variation in frequency of mothers screened positive for depressive symptoms**

**Residential region**			**N (%)**			**Univariate** ^**d**^		**Multivariate** ^**d**^	
**Description**	**Distance from the nuclear power plant** ^**a**^ **(km)**	**Average hourly radiation level in August 2011** ^**b**^ **(μSv/hr)**	**Total** ^**c**^	**Depressive symptoms**		**OR (95% CI)**	**P value**	**aOR (95% CI)**	**P value**
				**Positive**	**Negative**				
Kenchu (Middle region)	58	0.97	**2503 (30.5)**	682 (27.3)	1821 (72.8)	1.00		1.00	
Kenpoku (North region)	63	1.12	**2056 (25.1)**	618 (30.1)	1438 (69.9)	1.14 (1.01–1.31)	0.04	1.13 (0.99–1.29)	0.07
Kennan (South region)	81	0.45	**548 (6.7)**	150 (27.4)	398 (72.6)	1.00 (0.82–1.24)	0.95	0.98 (0.79–1.21)	0.83
Soso (Coastal region)	24	0.44	**851 (10.4)**	292 (34.3)	559 (65.7)	1.39 (1.18–1.65)	<0.01	1.36 (1.15–1.62)	<0.01
Iwaki (Coastal region)	43	0.18	**1316 (16.1)**	319 (24.2)	997 (75.8)	0.85 (0.73–1.00)	0.05	0.84 (0.71–0.98)	0.03
Aizu, Minamiaizu (Mountainous region)	98, 115	0.14, 0.07	**922 (11.3)**	201 (21.8)	721 (78.2)	0.74 (0.62–0.89)	<0.01	0.72 (0.60–0.87)	<0.01

^a^Distance between Fukushima Daiichi nuclear power plant and prefectural office branch in each region.

^b^Average of hourly monitoring for 31 days in August 2011, reported by the prefectural government.

^c^Column proportions are shown for the total distribution.

^d^Logistic regression analysis was used. Multivariate analysis controlled for maternal age (yr; aOR = 0.98, 95% CI = 0.97–0.99) and birth history (ref = 1 or more; aOR = 1.15, 95% CI = 1.03–1.28).

**Figure 2:**
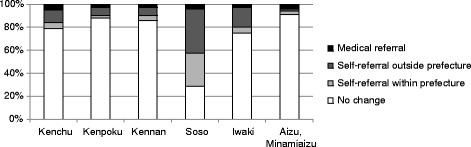
**Regional variations in the proportion of mothers that experienced obstetrical care interruption.**

The proportion of mothers that changed medical facilities was also highest in Soso (Figure 2). With regard to the effect that such a change had on depressive symptoms (Table 3), mothers that self-referred (voluntarily changed facilities following the disaster) to either another facility within Fukushima Prefecture or to one in a different region showed a significantly higher risk for a positive screen. Region-specific analysis revealed that adjusted risk was significantly higher for mothers that self-referred whilst living in less affected regions (self-referral within the prefecture: aOR = 1.46, 95% CI = 1.10–1.95, p = 0.01; self-referral outside the prefecture: aOR = 1.43, 95% CI = 1.16–1.76, p < 0.01; medical referral: aOR = 1.99, 95% CI = 0.73–1.37, p = 0.99). In addition, the more adversely affected coastal regions also reported a significantly higher risk among mothers that experienced medical referral (self-referral within the prefecture: aOR = 1.47, 95% CI = 1.10–1.96, p < 0.01; self-referral outside the prefecture: aOR = 1.37, 95% CI = 1.08–1.73, p = 0.01; medical referral: aOR = 2.76, 95% CI = 1.62–4.69, p < 0.01).

**Table 3:** **Obstetrical care and frequency of mothers who screened positive for depressive symptoms**

	**N (%)**			**Univariate** ^**b**^		**Multivariate** ^**b**^	
	**Total** ^**a**^	**Depressive symptoms**		**OR (95% CI)**	**P value**	**aOR (95% CI)**	**P value**
		**Positive**	**Negative**				
Changed medical facility after the disaster^c^							
No change	**6189 (77.4)**	1600 (25.9)	4589 (74.2)	1.00		1.00	
Self-referral within prefecture	**526 (6.6)**	176 (33.5)	350 (66.5)	1.44 (1.19–1.74)	<0.01	1.32 (1.07–1.63)	0.01
Self-referral outside prefecture	**999 (12.5)**	326 (32.6)	673 (67.4)	1.39 (1.20–1.60)	<0.01	1.29 (1.10–1.51)	<0.01
Medical referral	**286 (3.6)**	91 (31.8)	195 (68.2)	1.34 (1.04–1.73)	0.03	1.28 (0.98–1.67)	0.07

^a^Column proportions are shown for the total distribution.

^b^Logistic regression analysis was used. Multivariate analysis controlled for maternal age (yr; aOR = 0.98, 95% CI = 0.97–0.99), obstetrical complications before the disaster (ref = no; aOR = 1.35, 95% CI = 1.17–1.56), infant’s asphyxia (ref = no; aOR = 1.57, 95% CI = 0.99–2.47), and congenital anomaly (ref = no; aOR = 1.96, 95% CI = 1.46–2.62), along with residential region (ref = Kenchu; Kenpoku aOR = 1.16, 95% CI = 1.01–1.33; Kennan aOR = 0.95, 95% CI = 0.76–1.18; Soso aOR = 1.15, 95% CI = 0.95–1.39; Iwaki aOR = 0.81, 95% CI = 0.68–0.95; Aizu and Minamiaizu aOR = 0.77, 95% CI = 0.64–0.92).

^c^Fifty-two women who gave multiple reasons for referral and 134 women with unknown reason for facility change were excluded.

## Discussion

We found that a high proportion of mothers who were pregnant at the time of the Fukushima nuclear disaster exhibited depressive symptoms. This proportion was found to be higher among mothers in the region in which the damaged nuclear power plant is located and lower in regions that were less affected by the nuclear accident. In addition, a greater prevalence of depressive symptoms was reported among mothers that experienced an interruption in their obstetrical care following the disaster.

Both median maternal age (30 years) and the proportion of low birth weight infants (7.3%) were similar to the nationwide averages reported in the general census (31 years and 8.3% among singletons respectively) [15]. It may therefore be inferred that respondents did not differ considerably in these basic characteristics.

The proportion of positive screens in the present study was higher than that reported using the same measure in other regions of Japan. For example, in Mishina’s study of 475 mothers who attended a postpartum checkup in Osaka (eastern metropolitan area), 19.8% responded affirmatively at one month, and 1.4% was found to test positively at four months [16]. Postpartum depression, as measured by the EPDS, is an indicator used to evaluate the national maternal and child health plans and has a prevalence of approximately 10% according to a 2009 governmental report [17]. Among recent regional surveys in Japan that reported similar percentages using the EPDS, the Hamamatsu Birth Cohort reported a depression rate of 11% within one month, and 4% between 2 and 3 months postpartum [18]. Based on the positive and negative predictive values of the two-item screen measure (42% and 97%, respectively) [12], the estimated proportion of positive screens on the EPDS would be 14% among mothers in the present study. Considering the reported reduction in depression prevalence over time following birth, our finding that 27.6% of all mothers surveyed at around 6 months postpartum showed depressive symptoms is remarkably high. Following similar findings conducted after previous radiation disasters that indicate that mothers of young children are a high-risk group for mental health consequences [1,2], our results suggest that upgrading the level mental health support for mothers with infants should be considered a high priority in the acute phase of a nuclear disaster response.

The present findings also confirm the importance of established risk factors for depressive symptoms. As such, in this disaster cohort, younger maternal age, first-time motherhood, past abortion history, psychiatric history, obstetrical complications, and infant abnormalities were all significantly associated with depressive symptoms [18-21]. These findings indicate that, in a post-disaster setting, attention should still be paid to known risk factors for mental health problems. Suzuki and Weissbecker support this notion in their assertion that, immediately following a disaster, responses targeting mental health should be built on existing systems [22].

After controlling for potential confounders, our data showed that risk for depressive symptoms was the greatest in Soso, the region in which the affected nuclear power plant is located, and smallest in regions with lower radiation levels. This result is similar to previous findings of regional variation in mothers’ depressive symptoms within Fukushima City [9], and may be a reflection of mothers’ worries about radiation exposure. These findings are a cause for concern because previous studies have shown that maternal worry about radiation levels lingered for many years after the Chernobyl accident and was associated with poorer mental health [2]. It should be noted, however, that there is a general negative correlation between socioeconomic status and overall mental distress [23]. This may have significant bearings on the results of the present study, given that, according to the prefectural statistics of 2011 [24], Soso reported the lowest average per capita income level in the prefecture prior to the disaster. Specifically, where the prefectural average is set at 100, the average per capita income in Soso was 77.6, compared to 103.7 in Kenchu, 107.5 in Kenpoku, 103.5 in Kennan, 100.5 in Iwaki, 93.4 in Aizu, and 85.5 in Minamiaizu. Therefore, careful interpretation and further investigation are warranted regarding regional variations in the depressive states of new mothers.

Another factor of interest in the current study, interruption of post-disaster obstetrical care, significantly increased mothers’ risk for depressive symptoms. In general, continuity of obstetrical care is associated with better patient satisfaction [25]. In Japan, the tradition of Satogaeri Bunben (pregnant women returning to their parental home during the perinatal period), which is believed to provide better maternal support, has recently been shown to have no preventable effect on postpartum depression, due in part to negative influences of interruption of perinatal care [26]. In the present study, this effect was especially pronounced when mothers self-referred to a different facility following the disaster. A region-specific analysis further indicated that medical referral was also a significant risk factor in severely affected coastal regions. This finding is in line with a previous study of pregnant women affected by Hurricane Katrina, which found that maternal depression was associated with a loss of resources, including social support network and daily routine [27]. Another post-earthquake study from Japan reported that mothers’ EPDS score was associated with their satisfaction with delivery, including obstetrical care [28]. Moreover, an interruption in care could be accompanied by evacuation and other disaster-related incidents, which may further affect maternal mental health. Thus, mothers affected by a disaster may require interpersonal support to help restore psychosocial resources. Additionally, changes in obstetrical facilities could be practical indicators to help health care providers identify those mothers who are most in need.

Based on the above findings, we recommend that during the prioritization and resource allocation of mental health support, close attention be paid to regional variations in risk for mental health among new mothers. In the present study, the risk was elevated in a region nearest to the damaged nuclear power plant. Additionally, we recommend that mental health care providers pay careful attention to mothers whose antenatal care suffers interruption, such as those having to self-refer to a different facility, following a disaster.

### Limitations and strengths

There were two major methodological limitations in the current study. First, our main outcome measure was a conventional depression screen with only two items. Although its simplicity is a practical strength in screening, especially via a mail survey, it is likely that the true prevalence of diagnosable postpartum depression is lower than that reported here. Second, data on socioeconomic status, which may influence depressive symptoms, were not collected so as to avoid a culturally sensitive question and to shorten the questionnaire. For example, the national census collects information on educational levels only every ten years and the rate of incomplete information in the latest 2010 survey was 12% [15].

Strengths of the research include the extensive range of participants surveyed, because in the present study, all women who were pregnant during the post-disaster period were contacted by utilizing the national pregnancy registration, which is unique to Japan’s maternal and child health care system. Although obstetrical and infant medical histories were self-reported, mothers could refer to the Maternal and Child Handbook, which is provided to every pregnant woman in Japan at the time of their pregnancy registration, and is used among physicians and midwives to write medical records at every antenatal and postnatal visit, to aid in their reporting.

## Conclusion

The current analysis of the population-based FHMS among new mothers confirmed the necessity of providing mental health support to this well-known, high-risk group after a nuclear disaster. Such support efforts are recommended to address regional variations in mothers’ needs and their experiences of obstetrical care interruptions after the disaster.
